# Artificial intelligence for personalized management of vestibular schwannoma: a multidisciplinary clinical implementation study

**DOI:** 10.1093/jamiaopen/ooaf163

**Published:** 2026-01-06

**Authors:** Navodini Wijethilake, Steve Connor, Anna Oviedova, Marina Ivory, Rebecca Burger, Jeromel De Leon De Sagun, Amanda Hitchings, Ahmed Abougamil, Theofanis Giannis, Christoforos Syrris, Kazumi Chia, Omar Al-Salihi, Rupert Obholzer, Dan Jiang, Eleni Maratos, Sinan Barazi, Nick Thomas, Tom Vercauteren, Jonathan Shapey

**Affiliations:** School of Biomedical Engineering & Imaging Sciences, King’s College London, London SE1 7EH, United Kingdom; School of Biomedical Engineering & Imaging Sciences, King’s College London, London SE1 7EH, United Kingdom; King’s College Hospital NHS Foundation Trust, London SE5 9RS, United Kingdom; King’s College Hospital NHS Foundation Trust, London SE5 9RS, United Kingdom; School of Biomedical Engineering & Imaging Sciences, King’s College London, London SE1 7EH, United Kingdom; King’s College Hospital NHS Foundation Trust, London SE5 9RS, United Kingdom; King’s College Hospital NHS Foundation Trust, London SE5 9RS, United Kingdom; King’s College Hospital NHS Foundation Trust, London SE5 9RS, United Kingdom; King’s College Hospital NHS Foundation Trust, London SE5 9RS, United Kingdom; King’s College Hospital NHS Foundation Trust, London SE5 9RS, United Kingdom; King’s College Hospital NHS Foundation Trust, London SE5 9RS, United Kingdom; Guy’s and St Thomas’ NHS Foundation Trust, London SE1 9RT, United Kingdom; Guy’s and St Thomas’ NHS Foundation Trust, London SE1 9RT, United Kingdom; Guy’s and St Thomas’ NHS Foundation Trust, London SE1 9RT, United Kingdom; Guy’s and St Thomas’ NHS Foundation Trust, London SE1 9RT, United Kingdom; King’s College Hospital NHS Foundation Trust, London SE5 9RS, United Kingdom; King’s College Hospital NHS Foundation Trust, London SE5 9RS, United Kingdom; King’s College Hospital NHS Foundation Trust, London SE5 9RS, United Kingdom; School of Biomedical Engineering & Imaging Sciences, King’s College London, London SE1 7EH, United Kingdom; School of Biomedical Engineering & Imaging Sciences, King’s College London, London SE1 7EH, United Kingdom; King’s College Hospital NHS Foundation Trust, London SE5 9RS, United Kingdom

**Keywords:** vestibular schwannoma, deep learning, segmentation, feature extraction, personalized management

## Abstract

**Objectives:**

Management of patients with vestibular schwannoma (VS) relies on precise tumor size and growth trend evaluation. We introduce and evaluate a novel computer-assisted reporting tool for clinical decision support during multidisciplinary team meetings (MDTMs) for VS patients.

**Materials and Methods:**

Our approach exploits deep learning for tumor segmentation, automating tumor volume, and standard linear measurement extraction. We conducted 2 simulated MDTMs with the same 50 patients evaluated in both arms to compare our proposed approach against the standard process, focusing on its impact on preparation time and decision-making.

**Results:**

Automated reports provided acceptable information for an expert neuroradiologist in 72% of cases, while the remaining 28% required some revision with manual feature extraction. The segmentation models used in this report generation task achieved Dice scores of 0.9392 (±0.0351) for contrast-enhanced T1 and 0.9331 (±0.0354) for T2 MRI in delineating whole tumor regions. The automated computer-assisted reports that included additional tumor information initially extended the neuroradiologist’s preparation time for the MDTM (2 min 54 s [±1 min and 22 s] per case) compared to the standard preparation time (2 min 36 s (±1 min and 5 s] per case). However, the computer-assisted simulated MDTM approach significantly improved (*P* < .01) MDTM efficiency, with shorter discussion times per patient (1 min 15 s [±0 min and 28 s] per case) compared to standard simulated MDTM (1 min 21 s [±0 min and 44 s] per case).

**Discussion:**

An initial learning curve in interpreting new data measurements is quickly mastered and the enhanced communication of growth patterns and more comprehensive assessments ultimately provides clinicians with the tools to offer patients more personalized care.

**Conclusion:**

This pilot clinical implementation study highlights the potential benefits of integrating automated measurements into clinical decision-making for VS management.

## Introduction

Vestibular schwannoma (VS) is a benign brain tumor originating from myelinating Schwann cells within the vestibular division of the vestibulocochlear nerve. The incidence rate of VS is rising.^1^ In the United Kingdom, the incidence rate is approximately 2.2 per 100 000 people per year^2^ with some estimates indicating that approximately 1 in 1000 people will be diagnosed with a VS in their lifetime.^3^ The increasing availability and improved quality of magnetic resonance imaging (MRI) has resulted in higher proportion of small asymptomatic tumors now being diagnosed. For smaller tumors, expectant management with lifelong imaging is often advised^4^ with patients proceeding to stereotactic radiosurgery (SRS) or conventional open surgery should the tumor demonstrate growth on serial imaging. Even after treatment, patients typically require an extended period of surveillance.

The assessment of VS growth requires a standardized measurement approach.^5^ The 2001 Consensus Meeting on Reporting Results in Acoustic Neuroma recommends distinguishing intrameatal and extrameatal portions and measuring the largest extrameatal diameter.^6^ When a tumor is entirely intrameatal, this measurement becomes the maximum whole tumor diameter. For VS treatment decisions, both linear and volumetric measurements are applicable. As per the European Academy of Otology & Neuro-Otology position statement, the criteria for significant VS growth include a >2 mm increase in diameter, a >1.2 cm^3^ volume change, or a >20% volume change.^7^ The United Kingdom introduced cancer multidisciplinary team meetings (MDTMs) to ensure uniform high-quality care for all cancer patients, regardless of origin.^8^ Defining overall growth and growth rate is a key element in determining treatment strategies for patients with VS,^4^^,^^7^ alongside other factors such as absolute size, patient comorbidities, hearing, age, and patient preference. The manual extraction of linear measurements is a time-consuming process that is susceptible to variation and interobserver error.^9^ Volumetric measurements offer higher sensitivity and precision, but existing methods, including manual or semiautomated tumor segmentation, are labor-intensive, lack standardization and are prone to variability and subjectivity.^10^^,^^11^ Additionally, the lack of readily available standardized software has hindered the adoption of incorporating volumetric measurements into routine clinical practice.

Artificial intelligence (AI) has emerged as a valuable tool in various aspects of VS research. Deep learning and machine learning techniques have been utilized for tumor segmentation,^12–14^ growth prediction,^15^^,^^16^ surgical outcome prediction,^17^^,^^18^ and Koos grade prediction.^19^ Previous work has shown that AI tools possess the technical capability to fully automate the detection and segmentation of VS,^12^^,^^20^ and can also delineate the tumor’s intra- and extrameatal components.^14^ These tools formed the basis for automating the extraction process of both linear and volumetric measurements in this study. Nevertheless, while AI has shown promise in VS management, the assessment of its clinical applicability and its impact on decision-making requires further investigation.

**Figure 1.**
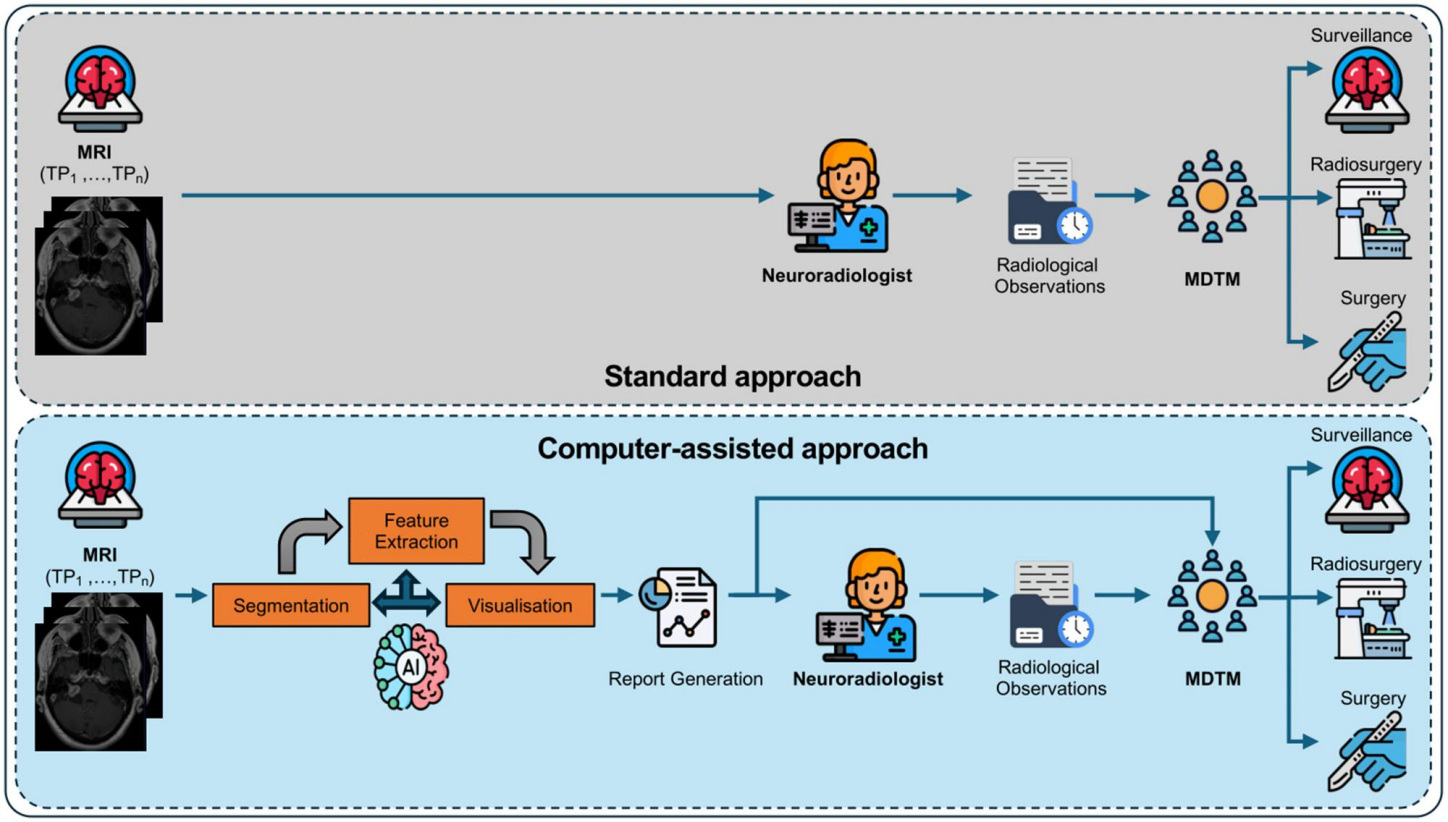
Overview diagram of our clinical implementation study, which consists of 2 arms to compare our proposed computer-assisted approach against the standard process, focusing on the personalized management of VS. In the computer-assisted approach, the automated report was utilized both during the neuroradiologist’s preparation for the simulated MDTM and during the MDTM itself, where it supported the decision-making process. Abbreviations: MDTM, multidisciplinary team meeting; VS, vestibular schwannoma.

In this study, we developed an automated imaging biomarker report generator that employs deep learning segmentation and computer algorithms for feature extraction and visualization. Each report included longitudinal measurements of the tumor, representative axial views of the tumor, and graphical representations to illustrate changes in both linear and volume measurements. This study assesses the overall process of implementing AI-driven automated reporting methods in a simulated controlled real-world context (Figure 1). To the best of our knowledge, this is the first study to integrate AI-based outcomes into clinical practice evaluating their impact on clinical decision-making for tumor management.

## Materials and methods

To evaluate the impact on standard clinical practice, 2 separate simulated MDTM sessions involving 50 referred patients were conducted in a controlled setting. Decisions made in the simulated MDTMs did not impact patient care. The first session adhered to the standard preparation procedure with manual extraction, while the second session introduced the automated imaging biomarker report.

### Datasets

For his retrospective exploratory study, we utilized 2 datasets: KCH MC-RC and UCLH MC-RC, each obtained from different referral centers in London, United Kingdom. UCLH MC-RC was used for internal segmentation model development (training, hyperparameter tuning, and testing), whereas KCH MC-RC was used for external validation during the CAS-MDTM process. Further details can be found in Appendix A.1.

#### Ethics statement

This study was approved by the NHS Health Research Authority and Research Ethics Committee (18/LO/0532). Because patients were selected retrospectively and the MR images were completely anonymized before analysis, no informed consent was required for the study.

### Formation of automated imaging biomarker reports

#### Deep learning model development

Building on the methodology in Wijethilake et al,^14^ we adopted a 2-stage approach employing the default 3D full-resolution UNet from the nnU-Net framework (referred to as 3D nnU-Net). Additional information regarding the internal training, hyperparameter tuning and testing sets, methodology and training procedures can be found in Appendices A.1 and A.2 (What we refer to as hyperparameter tuning set is often referred as validation set in the machine learning community. We use the terminology “hyper-parameter tuning” to avoid any ambiguity with regard to our independent external validation set.). There was no overlap in patients between the internal training dataset and the external validation set used in this study’s simulated MDTMs.

#### Feature extraction and visualization

For the KCH MC-RC dataset, we acquired segmentation masks using the best-performing deep learning models and utilized them in the feature extraction process. The primary linear measurement we extracted for each VS was the maximum tumor diameter. This measurement could be derived from 2 regions: the entire tumor region ($D^{WT}$) and/or the extrameatal region of the tumor ($D^{EM}$). When the extrameatal tumor portion was small or negligible, radiologists typically measured the linear dimension for the entire tumor region (combining both intra- and extrameatal regions).

More details about the linear features and selection of the appropriate measurement to present are provided in Appendix A.3. Further, in our previous study,^21^ we conducted a preliminary evaluation comparing automated and an expert’s manual linear measurements.

##### Choice of diameter to display to the clinical team

To streamline and simplify the presentation of data for the clinical team, we choose to extract and display only one of these diameters $D^{WT}$ and $D^{EM}$. The selection of which diameter to present to the users depends on the patient’s operative status and is influenced by the three additional features introduced earlier. For cases that have undergone surgery, we extract the maximum whole tumor diameter. For preoperative cases, we consider 2 subscenarios. The first subscenario occurs when the predicted mask is entirely intracanalicular, meaning only an intrameatal region is present. In this case, the intrameatal region represents the entire tumor, and we extract its maximum diameter. The second subscenario arises when the predicted mask includes an extrameatal portion. We then evaluate three additional features to determine which diameter should be extracted. In this second subscenario, the presented feature is decided using the following algorithm:

**IF**  $d_{\left(\mathrm{intra},∥\right)}$  ≥  $d_{\left(\mathrm{extra},∥\right)}$

   → use $D^{WT}$

**ELSE** 

  **IF**  $d_{\left(\mathrm{extra},⊥\right)}$  >2 mm

    → use $D^{EM}$

  **ELSE IF**  $d_{\left(\mathrm{extra},⊥\right)}$  ≤2 mm

    → use $D^{WT}$

Additionally, volume measurements were extracted for the intra- and extrameatal regions. Visualization of the axial slice with the maximum D was done using the 3D Slicer software extension with Python.^22^ Additionally, 3D volume mask of the tumor was visualized. Further, in our previous study,^21^ we conducted a preliminary evaluation comparing automated and an expert’s manual linear measurements.

#### Automated generation of the imaging biomarker report

**Figure 2.**
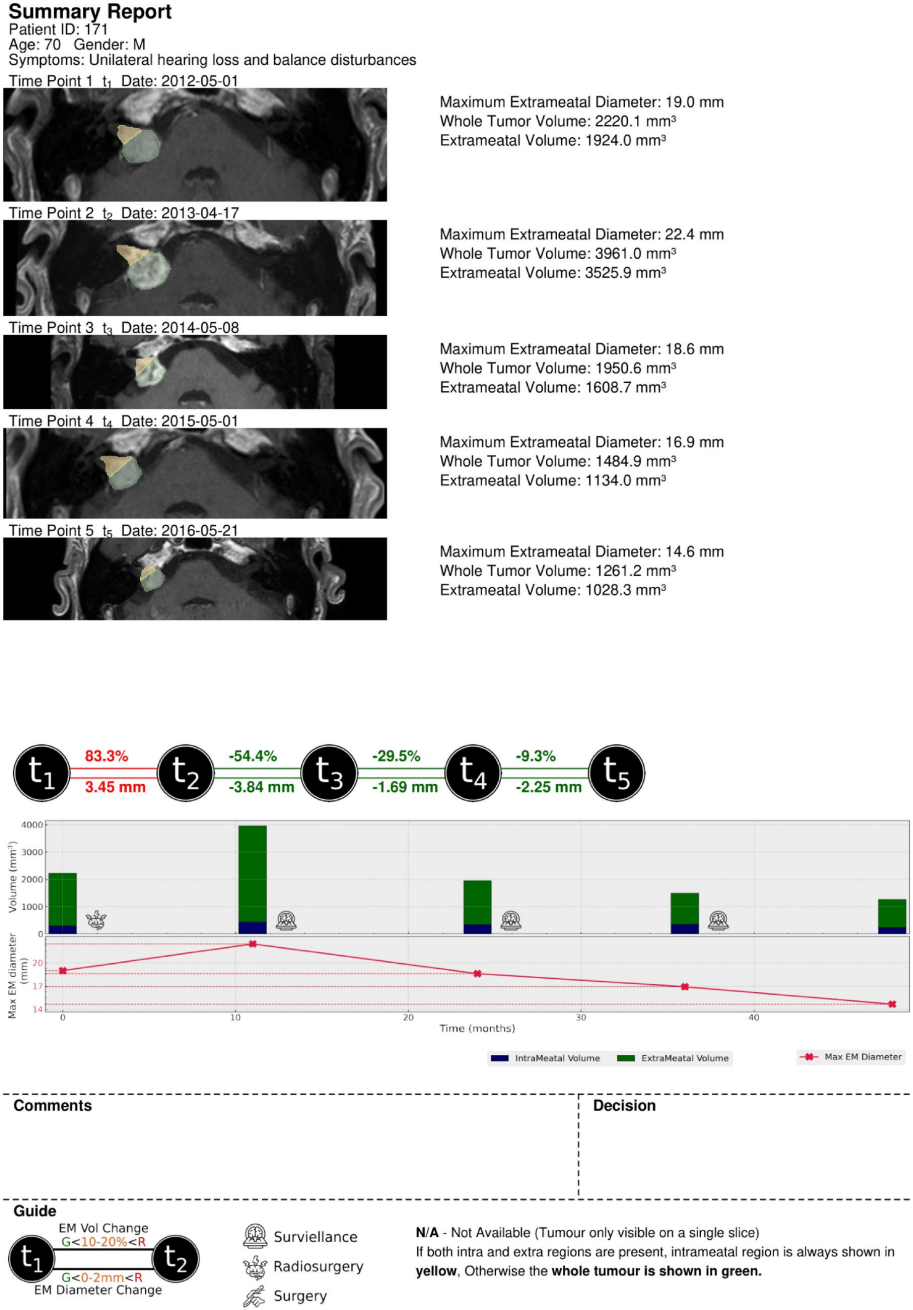
A summary report (this is an accepted report by the neuroradiologist).

Automated report generation followed 2 key steps: (1) segmentation and (2) feature extraction. For each patient a summary report and an extended version were generated. In the summary report, an MRI axial slice representative of each time point was included, along with the intra-/extrameatal mask, corresponding extrameatal and whole tumor volumes, and maximum D measurement. Volume measurements were not presented if the tumor was present in more than 1 axial slice. The summary report formation details, along with an example report, are provided in Figure 2. Additionally, an extended report is generated with all the measurements and 3D visualizations.

### Simulated MDTMs

#### Preparation for the standard MDTM

The neuroradiologist prepared for the SS-MDTM under time-constrained conditions to replicate clinical MDTM preparation following UK practice guidelines. Linear measurements were extracted from the index, second most recent, and most recent scans for each patient using the Picture Archiving & Communications System (PACS) workstation (Sectra workstation, Sectra AB, Sweden) at King’s College Hospital, London, United Kingdom. For each patient, the neuroradiologist recorded the absolute linear measurements, provided a qualitative description of the tumor, and made judgments regarding tumor growth (change/no change/equivocal) on a structured form. Longitudinal changes were assessed between the index and recent time points and between the second most recent and most recent time points. The starting and finishing times for manual feature extraction for each patient case were recorded. The neuroradiologist’s workload was also assessed using the NASA-TLX scoring system after completing the preparations, that evaluates a task based on 6 dimensions: mental demand, physical demand, temporal demand, performance, effort, and frustration, to provide an overall workload score.^23^ Higher NASA-TLX scores indicate greater perceived workload and stress associated with the task, reflecting increased cognitive or physical effort required by the user.

#### Execution of the standard MDTM

The SS-MDTM was conducted with the minimum required attendees, and it took place as a hybrid online-in-person meeting. The simulated skull-base MDTM included 2 skull-base neurosurgeons (N.T., J.S.), 2 clinical oncologists (K.C., O.A.), 2 clinical nurse specialists (A.H., J.D.L.D.S.), and 3 neurosurgical fellows (T.G., A.A., C.S.). Each case was presented to the MDTM by the MDTM coordinator (A.O). The neuroradiologist (S.C.) then presented the tumor measurements and observations, with the tumor displayed on the PACS system through screen sharing. The starting and finishing times of the MDTM were recorded.

#### Preparation for the CAS-MDTM

The CAS-MDTM was conducted 35 days after the SS-MDTM. To reduce the potential for bias, the order of the 50 cases was altered and randomly assigned. Once again, the neuroradiologist prepared for the CAS-MDTM in a time-constrained environment, this time utilizing the automated report. In this preparation phase, the neuroradiologist evaluated and decided on the acceptance or rejection of automated biomarkers for each patient’s MRI session (time point). This external validation was performed considering the segmentation outcomes provided by the deep learning model, while assessing the robustness of the deep learning-based segmentation model. If a session was rejected, the neuroradiologist manually extracted the linear measurements. The assessments conducted during this process followed the same procedure as in the SS-MDTM, utilizing the index, second most recent, and their most recent scans. The overall growth from the index scan or interval growth from the second most recent scan was documented.^7^

#### Execution of the computer-assisted MDTM

The CAS-MDTM followed the same format and impact assessment criteria as the SS-MDTM but introducing an evaluation of volume differences between the scans. CAS-MDTM was held 35 days after the SS-MDTM. Automated reports were distributed to all participants prior to the MDTM and the neuroradiologist (S.C.) presented his observations, referencing either linear or volume measurements as necessary. During the CAS-MDTM, members also referred to the automated report to assist their decision-making.

## Results

### Deep learning-based segmentation

The segmentation model provided whole tumor Dice scores of 0.8962 (±0.0510) for T2 and 0.8655 (±0.0836) for T1C on the internal testing set after first stage of 2-stage framework. After 2-stage segmentation, the model achieved whole tumor Dice scores of 0.9392 (±0.0351) for T2 and 0.9331 (±0.0354) for T1C, respectively, on the internal testing set. The performance of the segmentation models during the internal testing phase is presented in Appendix B.1.

### Sample reports generated through automated imaging biomarker reporting

The report included a visual representation of volume and maximum diameter (D) change, using color coding to indicate growth (red), equivocal growth (orange), and no growth (green). Intra-/extrameatal volumes were displayed in a bar plot, and diameters in a line plot with the *x*-axis representing time in months. Icons on the graph represented the decisions made after each session, with a guide provided at the bottom of the report. Figure 2 illustrates a report generated using our proposed methodology. It also demonstrates a case where the tumor shows an initial increase in size following SRS, a known posttreatment effect that does not necessarily indicate treatment failure or disease progression.

### Neuroradiological preparation for the CAS-MDTM

In 72% of cases (36 patients), the automatically generated segmentations and linear/volume measurements were accepted and used for assessing tumor growth by the neuroradiologist (see “Sample Reports Generated Through Automated Imaging Biomarker Reporting”). The neuroradiologist’s observations, combining manual and automated measurements, were presented to the MDTM and in 12 cases the neuroradiologist chose to *only* present the automatically generated volume measurements provided in the summary report. For 16% of cases (8 patients), 1 session had unacceptable segmentation (Appendix B.3). In these cases, the neuroradiologist manually extracted the linear measurements for the rejected session.

In the remaining 12% of cases (6 patients), the automated outcomes required complete revision by the neuroradiologist during the preparation of the CAS-MDTM. This occurred when at least 2 sessions had unacceptable segmentations or mismatching linear measurements (Appendix B.4). In such instances, only the neuroradiologist’s manual extractions were used in the MDTM.

Both the SS-MDTM and CAS-MDTM preparations placed a “somewhat high” workload on the neuroradiologist with CAS-MDTM preparation having a higher workload compared to SS-MDTM preparation (NASA-TLX [NASA Task Load Index] 45.5 vs 48, cf “Simulated MDTMs” in “Methods” section).

### Simulated MDTMs

In the SS-MDTM, 7/50 patients were referred for active treatment including 5 for SRS and 2 for surgery. Thirty-nine patients were placed under surveillance and 4 were discharged. Patients under surveillance were imaged in line with our published protocol.^4^

In the CAS-MDTM, 6 patients were referred for active treatment discussions, including 5 for SRS and 1 for surgery. Thirty-eight patients were placed under surveillance and 6 were discharged. In 2/50 patients, a surgical intervention was recommended in the SS-MDTM, while it was only recommended in 1/50 patients in the CAS-MDTM.

**Figure 3.**
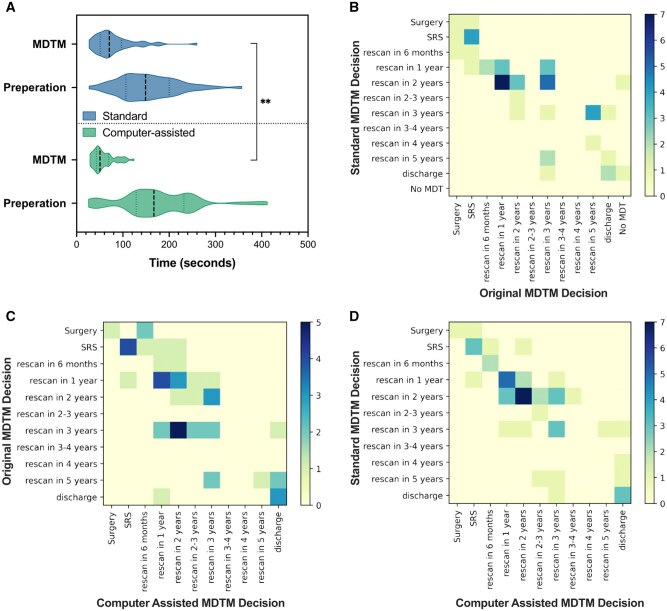
(A) Comparison of the timings of radiology preparation and MDTM for standard and computer-assisted approaches. **P<.01. (B) Confusion matrix between the SS-MDTM decisions and the (actual) original MDTM decision. (C) Confusion matrix between the (actual) original MDTM decisions and the CAS-MDTM decisions. (D) Confusion matrix between the SS-MDTM and the CAS-MDTM decisions. Abbreviations: CAS-MDTM, computer-assisted simulated multidisciplinary team meeting; SS-MDTM, standard simulated multidisciplinary team meeting.

The average times for preparation and MDTM for both standard and computer-assisted approaches are presented in Table 1 with the distribution visualized in Figure 3B.

**Table 1.** Comparison of preparation and MDTM process durations in SS-MDTM and CAS-MDTM sessions.

		Average time per case	Total time	
Preparation	SS-MDTM	2 min 36 s (±1 min and 5 s)	2 h 7 min 30 s	n/s
	CAS-MDTM	2 min 54 s (±1 min and 22 s)	2 h 25 min 24 s	
MDTM	SS-MDTM	1 min 21 s (±0 min and 44 s)	1 h 2 min 36 s	^a^
	CAS-MDTM	1 min 15 s (±0 min and 28 s)	48 min 24 s	

Abbreviations: CAS-MDTM, computer-assisted simulated multidisciplinary team meeting; n/s, no significant difference; SS-MDTM, standard simulated multidisciplinary team meeting.

a *P*<.01.

## Discussion

In this study, we introduce a novel approach, CAS-MDTM, that integrates automated imaging biomarkers reports into VS clinical management. We validate this concept in a controlled simulated clinical setting, involving a multidisciplinary team of clinical experts. Through deep learning-based automated segmentation, we provide highly sensitive volume measurements, crucial for analyzing VS growth. This approach has the potential to streamline decision-making, enhancing clinical efficiency, and offers potential for more personalized patient care. The use of automated reporting resulted in a substantial improvement in clinical efficiency and the potential to enhance patient management quality.

To the best of our knowledge, our research represents the first clinical deployment of an AI supported report for tumor management, where we examine its effect on clinical workflow and decision-making process. Our study complies with the Checklist for Artificial Intelligence in Medical Imaging.^24^ Hawkins et al^25^ recently implemented a machine learning-based clinical workflow to perform segmentation, volumetric calculations, and generate reports using longitudinal MRI scans for low-grade glioma. However, their study did not explore how this technology could be integrated into clinical decision-making.

During preparations for the CAS-MDTM, the neuroradiologist manually extracted linear measurements when the whole tumor segmentation was unacceptable at any of the 3 time points (index, second most recent, and most recent).

**Figure 4.**
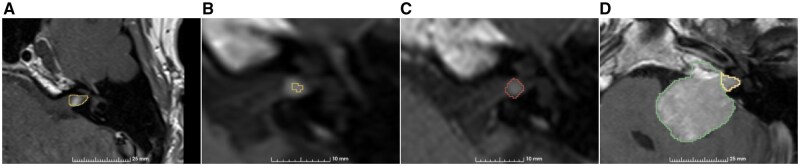
Examples of deep learning-generated segmentations rejected by the neuroradiologist. (A) Oversegmented slice. (B) Undersegmented slice. (C) Missed tumor detection. The manual annotation of the tumor region is shown in red. (D) Oversegmented and the incorrect intra-/extrameatal boundary.

We identified 4 scenarios contributing to faulty segmentations (Figure 4). Figure 4A visualizes a session with an oversegmentation of a small tumor, while Figure 4B and C depicts instances of undersegmentation and nonrecognition by the segmentation model, respectively. In such instances, the neuroradiologist manually extracted the linear measurements for these cases. Additionally, Figure 4D is an example of a session that was oversegmented, thus failing to provide an acceptable intra-/extrameatal boundary.

During SS-MDTM preparations, the neuroradiologist chose between $D^{WT}$ and $D^{EM}$, based on whether the maximum extrameatal dimension appeared larger than the porus on axial images. If the neuroradiologist was seeking serial comparable dimensions and the initial tumor was intrameatal, subsequent scans were interpreted using whole tumor volume measurements, even in the presence of a significant extrameatal component. This posed a significant challenge in the context of the automated imaging biomarker report, as we selected $D^{EM}$ according to our algorithm (Appendix A.3).

**Figure 5.**
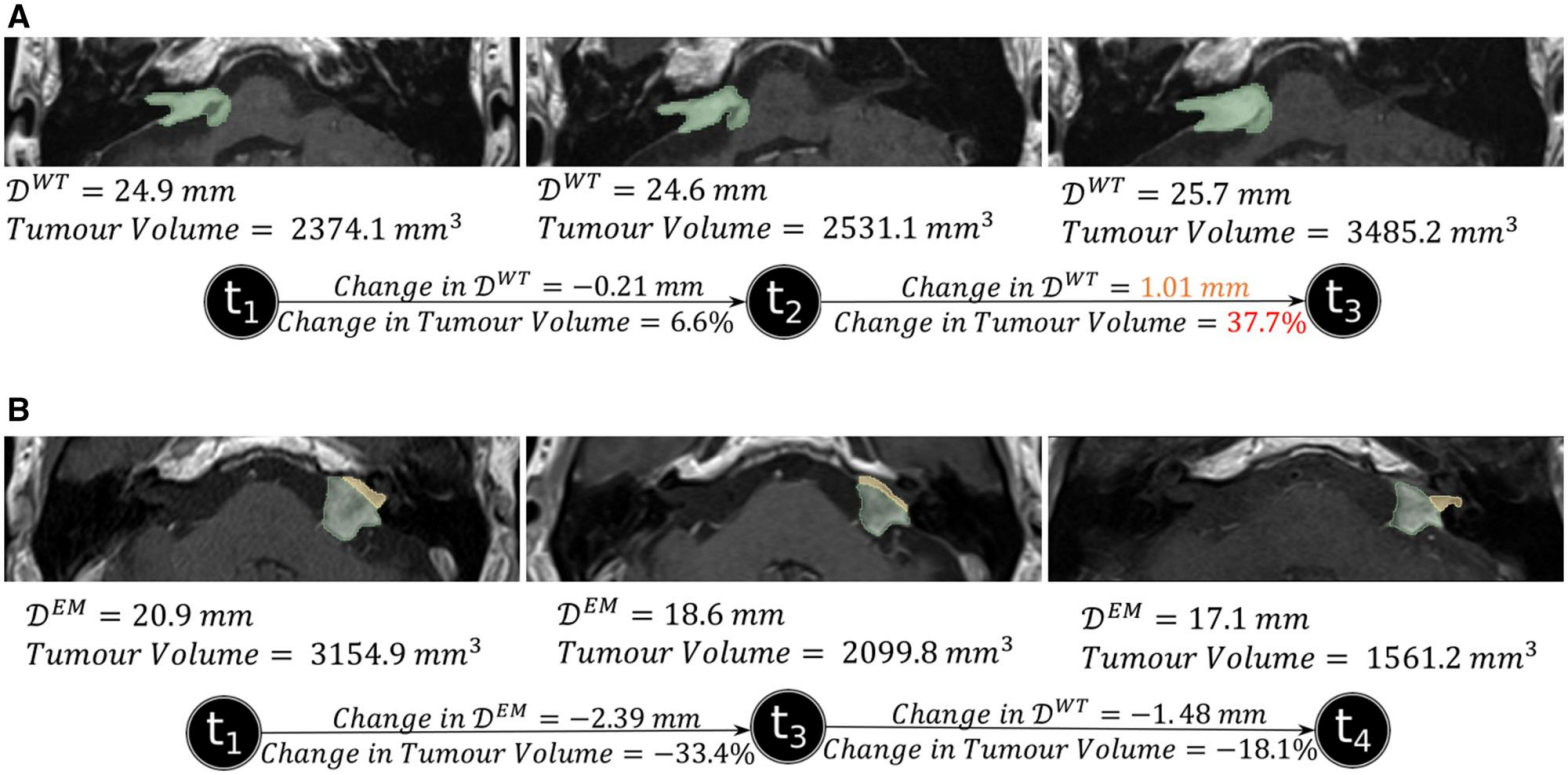
Two patient cases that depicts the importance of volume measurements. (A) A patient case where the volume measurement change presents a definitive growth in contrast to the linear measurement. (B) A patient case with an inconsistent and irregular intra-/extrameatal boundary, for which the tumor volume is more reliable.

For several cases, the whole tumor volume tended to provide more reliable observations on growth, as shown in Figure 5. Figure 5A displays postoperative yearly scans between 2011 and 2013, along with corresponding $D^{WT}$ and whole tumor volume measurements. The change between the second and third time points indicates definitive growth (a change in volume of over 20%), while the change in linear measurement ($D^{WT}$) suggests equivocal growth.

Differences in tumor boundaries between longitudinal scans was another reason for choosing whole tumor volume as the comparative metric for some patients. Figure 5B presents longitudinal scans of a post-SRS patient from 2012, 2016, and 2019 who had undergone SRS in 2005. Due to the irregular shape of the tumor, the separation of the intra-/extrameatal regions does not accurately depict the tumor’s behavior over time. These observations were highlighted by the neuroradiologist during the preparations and also when presenting to the MDTM.

Contrary to expectations, our results demonstrated that the average preparation time for the CAS-MDTM was slightly longer than for the SS-MDTM (not statistically significant). This was partly due to the learning curve experienced by the neuroradiologist, which might have been mitigated through training on a separate trial cohort. Moreover, when preparing for the CAS-MDTM, the neuroradiologist was assessing the validity of both the tumor segmentations and linear/volume measurements, which was different to the SS-MDTM preparations that exclusively utilized linear measurements. The information overload on the summary report may have also contributed to the extended preparation time and the high preparation workload in the CAS-MDTM.

The neuroradiologist’s preparation may also have been negatively affected by poor performance of the segmentation model. This may be a result of various factors including limited availability of the desired MRI sequences, nonstandard MRI acquisition, case complexity, tumor heterogeneity, the presence of very small tumors or residual tumor following surgery. Better automated segmentation results are likely to be achieved through the standardization of MRI sequences used to image VS and/or diversification of the training dataset. Nevertheless, minor adjustments will almost certainly be required in some difficult cases so an integrated interactive segmentation module, allowing the neuroradiologist to make real-time adjustments in the segmentation masks, should be developed before such technology can be fully integrated into the routine clinical workflow. Integrating such a module into PACS would further reduce the cognitive load of processing/visualizing various metrics, enabling the neuroradiologist to filter and focus on a specific preferred metric.

The total number of discharge decisions were higher in the CAS-MDTM (6 patients) compared to the SS-MDTM (4 patients). The clinical multidisciplinary team (MDT) agreed to discharge 3 of the same patients in both MDTMs. One patient discharged from the SS-MDTM was not discharged from the CAS-MDTM; 3 additional patients were discharged from the CAS-MDTM. Discharged patients typically included those in old age (>80 years) with small stable tumors. Figure 3C summarizes the similarities and differences between the SS-MDTM and the CAS-MDTM.

In the SS-MDTM, of 39 patients for whom surveillance imaging was originally recommended for actual patient care, the rescan interval increased for 11 patients (28.2%) decreased for 8 patients(20.5%), and remained unchanged for 16 patients (41%) as compared with the MDTs original clinical decision. Figure 3B illustrates how decisions taken in an SS-MDTM can change over time based on the original decisions made and the simulated MDTM decisions for the 50 patients. Of note, in the 3 patients where there was a discrepancy in the recommendation for treatment between the 2 MDTMs, there was only a difference in the determination of tumor growth in 1 patient. Patient management may have changed since the patient’s original clinical MDTM due to evolving clinical management and variations in the composition of MDTM participants together with their individual biases toward different treatments.

The use of computer-assisted reporting enabled the MDT to offer patients a personalized management approach beyond standard surveillance protocols. Decision-making in both SS-MDTM and CAS-MDTM was primarily based on tumor size measurements. However, other factors such as the patient’s age and treatment history significantly influenced the decisions. In future studies, further exploration of these decision discrepancies is also essential to better understand how clinical judgment, patient-specific factors, and AI-assisted insights interact to influence management choices.

Qualitative feedback obtained from the MDT demonstrated that the graphical representations illustrating growth trends over patients’ entire surveillance program were immensely beneficial. The inclusion of additional volume data played a vital role in enhancing the precision of interval change assessments particularly when deciding whether or not to extend the interval between surveillance scans. Oncologists especially valued having whole tumor volume measurements available as this is the metric used when delivering radiotherapy—not just the extrameatal portion.

From the neuroradiologist’s perspective, the full automated imaging biomarker report, with its multiple inputs (linear and volume measurements for every imaging timepoint), was more challenging to interpret compared to the standard approach, where only index, second most recent, and most recent imaging were used. Consequently, in the CAS-MDTM, observations were limited to the same 3 sessions to reduce complexity. Additionally, the neuroradiologist highlighted the importance of keeping an expert human-in-the-loop for the absence of thorough data verification may lead to clinicians misinterpreting critical information. Accordingly, the integration of AI-assisted tools into clinical workflows must be approached with a human-in-the-loop framework, where neuroradiologists and clinicians can interactively refine model outputs and provide oversight in complex cases. This approach will ensure that automated segmentation methods remain both reliable and adaptable to real-world clinical scenarios and also foster trust among clinicians, ensuring that these tools effectively support decision-making rather than introducing new sources of uncertainty and AI hallucinations. In both simulated MDTMs, the neuroradiologist also provided observations on additional radiological features such as cystic changes, peritumoral edema, and contrast enhancement patterns. While tumor size and volume are key parameters in monitoring vestibular schwannomas, these qualitative features are equally important for a comprehensive assessment. Their evaluation underscores the continued value of expert neuroradiological interpretation, which offers clinical insights beyond the scope of current automated algorithms. This highlights the future need to incorporate quantitative biomarkers and visualizations capable of capturing a broader spectrum of tumor and brain abnormalities.

We organized an online Patient Involvement focus group in partnership with the British Acoustic Neuroma Association to explore how patients could utilize this technology. We selected 12 individuals from a pool of over 200 interested patients across the United Kingdom, ensuring diversity in age, gender, and treatment experiences.

**Figure 6.**
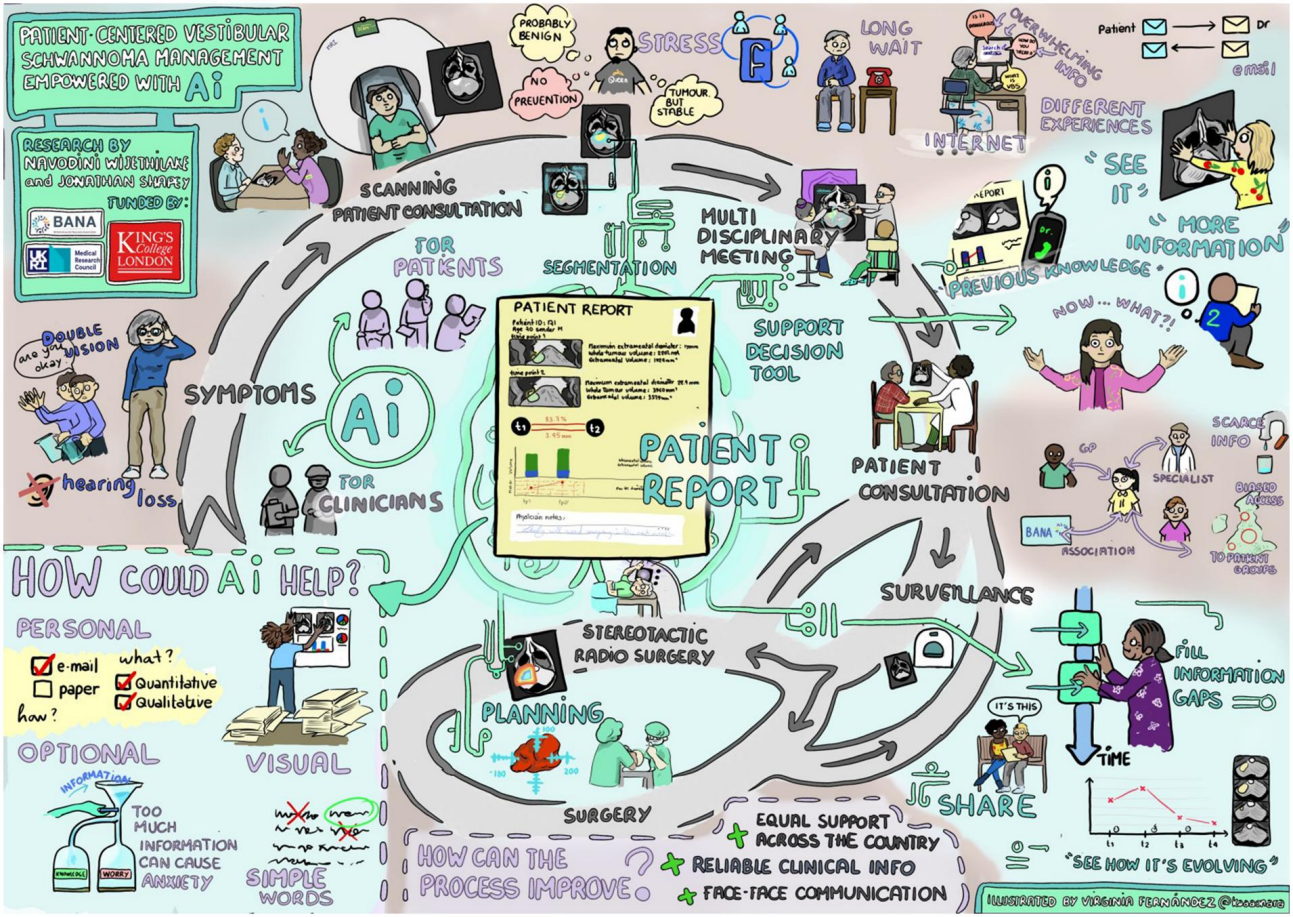
Visual illustration from the patient involvement session.

This session provided valuable insights into patient preferences for automated reports, emphasizing the importance of visual and personalized formats. Patients highlighted the significance of clarity and simplicity in reporting. An artistic representation visually depicted the event, referencing the key components highlighted by the patients (Figure 6).

The session highlighted how this report could be extended for use in combined MDTM clinics, as discussed in Colombo et al,^26^ where patients can access their personalized reports. This could improve workflow efficiency, patient-reported outcomes, decision transparency, and patient mental health.

In conclusion, this is the first study to report the clinical deployment of an AI-supported reporting tool for tumor management. We introduced computer-assisted automated reporting exploiting deep learning-based segmentation to aid VS management and evaluated its impact in a simulated clinical environment. This work demonstrated a significant improvement in the efficiency of clinical MDTMs and highlights the positive impact computer-assisted reporting could have in delivering more personalized patient care.

## Supplementary Material

ooaf163_Supplementary_Data

## Data Availability

The datasets used for the internal development of the segmentation models are openly available on the Cancer Imaging Archive at https://www.cancerimagingarchive.net/collection/vestibular-schwannoma-mc-rc/. The code for the automated report generation framework discussed in this paper is publicly available on GitHub at: https://github.com/navodini/AutomatedReportGenerationVS.
