# Supplementary material for: Artificial intelligence for personalized management of vestibular schwannoma: a multidisciplinary clinical implementation study
Source: JAMIA Open. 2026 Jan 6;9(1):ooaf163. doi: 10.1093/jamiaopen/ooaf163 (PMC12772638; doi:10.1093/jamiaopen/ooaf163)
Supplement: ooaf163_Supplementary_Data [file ooaf163_supplementary_data.zip › SimulatedMDM_JAMIA_Supplementary.pdf]

Appendix A Supplementary Methods

A.1 Datasets

We employed two datasets, KCH MC-RC and UCLH MC-RC, from separate referral centers in London, UK. UCLH MC-RC supported internal segmentation model development, while KCH MC-RC was used for external validation during the CAS-MDTM process. The flow of data and the inclusion/exclusion criteria are shown in Fig. A1.

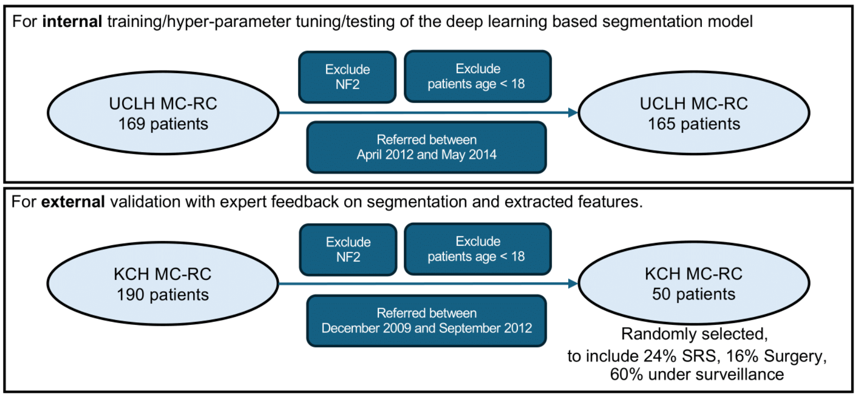

Fig. A1: Flow of cases in the two datasets we occupied for this study.

KCH MC-RC dataset for external validation during the CAS-MDTM process. The source cohort for this retrospective exploratory study consisted of patients referred to King's College Hospital, London, UK , between December 2009 and September 2012, aged over 18, with unilateral VS, excluding patients with neurofibromatosis type 2 (NF2).

Fifty patients were randomly selected for our study, with consecutive time points spanning 6 months to 11 years between the first and most recent scan (January 2012 - March 2021). Table A1 summarises the demographics of the selected cohort of 50 patients.

UCLH MC-RC dataset for internal development of the deep-learning models. To train an automate tumour delineation algorithm, we utilised our open-access, de-identified UCLH MC-RC dataset available on TCIA (The Cancer Imaging Archive) https://www.cancerimagingarchive.net/collection/vestibular-schwannoma-mc-rc/, which comprises MRI scans from 165 patients with unilateral VS [1]. Whole tumour annotation was conducted iteratively by a specialist MRI labeling company, Neuromorphometrics (Somerville, Massachusetts, USA). This annotation was then reviewed and validated by a panel of clinical experts, including a consultant neuroradiologist and a consultant neurosurgeon with the inter- and intrarater variability analysis, with an analysis of Inter- and intra-observer reliability.

Table A1: Demographics of the KCH MC-RC patient cohort consists of 50 patients used in the simulated MDTM.

**Category**	**N**	**Mean**	**SD**
**Age**	50	64.62	12.84
**Gender**			
Female	26 (48%)		
Male	24 (52%)		
**Monitoring period**	50	4.8 (yrs)	2.74 (yrs)
**Longitudinal scans per patient**	187	3.71 (scans)	1.35 (scans)
**Slice thickness of MRI scans**			
T2	42	1.20 (mm)	1.48 (mm)
Contrast enhanced T1	145	2.99 (mm)	0.561 (mm)
**Standard Tumour size measurement in the index scan**			
Extrameatal Diameter	25 (50%)	18.52 (mm)	9.04 (mm)
Whole tumour Diameter*	25 (50%)	10.36 (mm)	5.84 (mm)
**Interventional treatment History (prior to the most recent scan)**			
Surgery	8 (16%)		
SRS	12 (24%)		

* exclusively intrameatal tumours or post-operative cases

Table A2: Distribution of data between internal training, hyper-parameter tuning and testing sets during segmentation model development. N_p: Number of patients, N_s: Number of scans

Modality	Train		Valid		Test	
	N_p	N_s	N_p	N_s	N_p	N_s
T1C	41	53	6	8	12	15
T2	97	212	17	35	30	61

A detailed explanation of this process may be found in our previous work [1]. Subsequently, for cases with segmentations of either T1C or T2-weighted MRI modalities, intra-/extra-meatal segmentation (split segmentation) was performed by an expert neurosurgeon using MRtrix3 and ITK-SNAP tools [2, 3].

This dataset was used for internal training, hyper-parameter tuning, and testing of the segmentation model. Following this, the segmentation model was utilized during the CAS-MDTM process where the segmentation model was further externally validated on the KCH MC-RC dataset. The development of models was conducted independently for the T1C and T2 modalities.

Ethics statement. This study was approved by the NHS Health Research Authority and Research Ethics Committee (18/LO/0532). Because patients were selected retrospectively and the MR images were completely anonymised before analysis, no informed consent was required for the study.

A.2 Deep learning model development

Based on the approach detailed in [4], we implemented a two-stage methodology utilising the default 3D full-resolution UNet from the nnU-Net framework, along with the pre-processing steps, denoted as 3D nnU-Net [5]. In sequence, these stages aimed to achieve whole tumour and intra-/extra-meatal segmentation. For stage 2 training, we generated whole tumour masks through a 5-fold cross-validation process carried out in stage 1. In both stages, a loss function that combines Cross-entropy and Dice score was utilised. The model was trained using the internal training and hyper-parameter testing sets [1] and the performance was evaluated on the internal testing set. There was no overlap in patients between the internal training dataset and the external validation set used in this study's simulated MDTMs.

The best performing models were identified by considering the Dice score of each fold and were then used to obtain the segmentations of the KCH MC-RC dataset, which were later externally validated during the simulated CAS-MDTM. Only the first stage of the two-stage nnUNet framework was employed to obtain masks for the post-operative sessions in the KCH MC-RC dataset. The robustness of the deep learning based segmentation model outcomes were assessed by the expert neuroradiologist, during the MDTM preparations.

A.3 Extraction of the linear measurements

Selection of the posterior petrous pyramid axial plane. The first step to define and extract the relevant linear measurements is to identify the best axial plane to visualise the posterior petrous pyramid. For this purpose, we iterate through all axial planes containing both an intra- and extra-meatal tumour component according to our DL model output. We find the plane containing the largest distance between any intra- and extra-meatal points. We define this plane as the *posterior petrous pyramid axial plane.*

Extraction of linear measurement features in the posterior petrous pyramid axial plane. In this posterior petrous pyramid axial plane, we extract a linear segment that separates the intra- and extra-meatal component well, acknowledging that the DL model may lead to a slightly curved boundary between the intra- and extra-meatal components. We refer to this segment as the *meatal boundary segment* and define it by finding the most distant points on the boundary between the intra- and extra-meatal component as illustrated in Fig. A2(A).

From the posterior petrous pyramid axial plane, we extract three additional linear measurement features:

---

[1] What we refer to as hyper-parameter tuning set is often referred as validation set in the machine learning community. We use the terminology "hyper-parameter tuning" to avoid any ambiguity with regards to our independent external validation set.

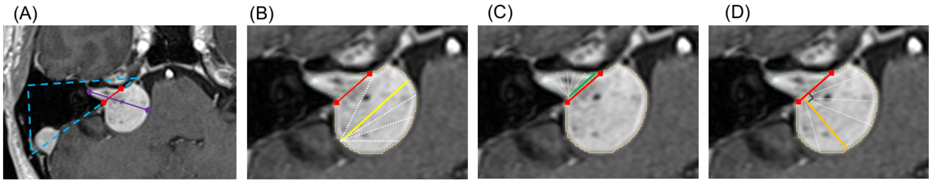

Fig. A2: (A) The posterior petrous pyramid region is shown in blue dashed margin and this **posterior petrous pyramid axial plane** is identified by considering the plane with largest distance between any intra- and extra-meatal points as shown in purple. The **meatal boundary segment** which that separates the intra-/extra-meatal regions is shown in red. (B) $\mathbf{d}_{(extra,\|)}$ is shown yellow (C) $\mathbf{d}_{(intra,\|)}$ is shown in green. (D) $\mathbf{d}_{(extra,\perp)}$ is shown in orange.

- The maximum intrameatal distance parallel to the meatal boundary segment: $\mathbf{d}_{(intra,\|)}$
- The maximum extrameatal distance parallel to the meatal boundary segment: $\mathbf{d}_{(extra,\|)}$
- The maximum extrameatal distance perpendicular to the meatal boundary segment: $\mathbf{d}_{(extra,\perp)}$

The parallel measurement to the meatal boundary segment $\mathbf{d}_{(intra,\|)}$ (resp. $\mathbf{d}_{(extra,\|)}$) was determined by iterating through all pairs of points on the contour of the intra-meatal (resp. extra-meatal) component, identifying those that define a segment parallel to the meatal boundary segment, and keeping the largest such segment as illustrated in Fig. A2(C) (resp. Fig. A2(B)).

Similarly, for the perpendicular measurement $\mathbf{d}_{(extra,\perp)}$, we iterate through all pairs composed of a point on the meatal boundary segment and a point on the contour of the extra-meatal component, identifying those pairs defining a segment perpendicular to meatal boundary segment, and keeping the largest such segment as illustrated in Fig. A2(D).

Extraction of the largest tumour diameter and largest extra-meatal region diameter. Independently of the previous linear features, we extract the largest tumour axial diameter $\mathcal{D}^{WT}$ and the largest extra-meatal region axial diameter $\mathcal{D}^{EM}$ across all axial planes. For efficiency, we iterate over all axial planes containing the tumour (resp extra-meatal component), employ the `find_boundaries` function from sklearn to extract the contour of the region, compute the convex hull using Andrew's monotone chain algorithm [6], and then determine the region diameter in the axial plane using the rotating caliper method. Finally, we keep the largest such axial diameter. We note that $\mathcal{D}^{WT}$ and $\mathcal{D}^{EM}$ may be found in distinct planes and that these planes may also be different than the posterior petrous pyramid axial plane defined above.

Appendix B Supplementary Results

B.1 Deep learning based segmentation

The segmentation model provided whole tumour Dice scores of 0.8962 (± 0.0510) for T2 and 0.8655 (± 0.0836) for T1C on the internal testing set after first stage of two-stage framework. After two-stage segmentation, the model achieved whole tumour Dice scores of 0.9392 (± 0.0351) for T2 and 0.9331 (± 0.0354) for T1C, respectively, on the internal testing set. Table B3 summarises the quantitative results of the segmentation conducted on the testing set of the internal UCLH MC-RC dataset.

Table B3: Quantitative results of the segmentation task, for intra-/extra-meatal segmentation and whole tumour segmentation on the internal testing cohort.

MRI	Case	Intrameatal	Extrameatal	Whole Tumour
T2	Stage 1	-	-	0.8962 (± 0.0510)
	Stage 2	0.8345 (± 0.0747)	0.8602 (± 0.2116)	0.9392 (± 0.0351)
T1	Stage 1	-	-	0.8655 (± 0.0836)
	Stage 2	0.8367 (± 0.0740)	0.7877 (± 0.2975)	0.9331 (± 0.0354)

References

[1] Kujawa, A., Dorent, R., Connor, S., Thomson, S., Ivory, M., Vahedi, A., Guilhem, E., Wijethilake, N., Bradford, R., Kitchen, N., *et al.*: Deep learning for automatic segmentation of vestibular schwannoma: a retrospective study from multi-center routine MRI. Frontiers in Computational Neuroscience **18**, 1365727 (2024)

[2] Tournier, J.-D., Smith, R., Raffelt, D., Tabbara, R., Dhollander, T., Pietsch, M., Christiaens, D., Jeurissen, B., Yeh, C.-H., Connelly, A.: MRtrix3: A fast, flexible and open software framework for medical image processing and visualisation. Neuroimage **202**, 116137 (2019)

[3] Yushkevich, P.A., Piven, J., Cody Hazlett, H., Gimpel Smith, R., Ho, S., Gee, J.C., Gerig, G.: User-guided 3D active contour segmentation of anatomical structures: Significantly improved efficiency and reliability. Neuroimage **31**(3), 1116–1128 (2006)

[4] Wijethilake, N., Kujawa, A., Dorent, R., Asad, M., Oviedova, A., Vercauteren, T., Shapey, J.: Boundary distance loss for intra-/extra-meatal segmentation of vestibular schwannoma. In: Machine Learning in Clinical Neuroimaging: 5th International

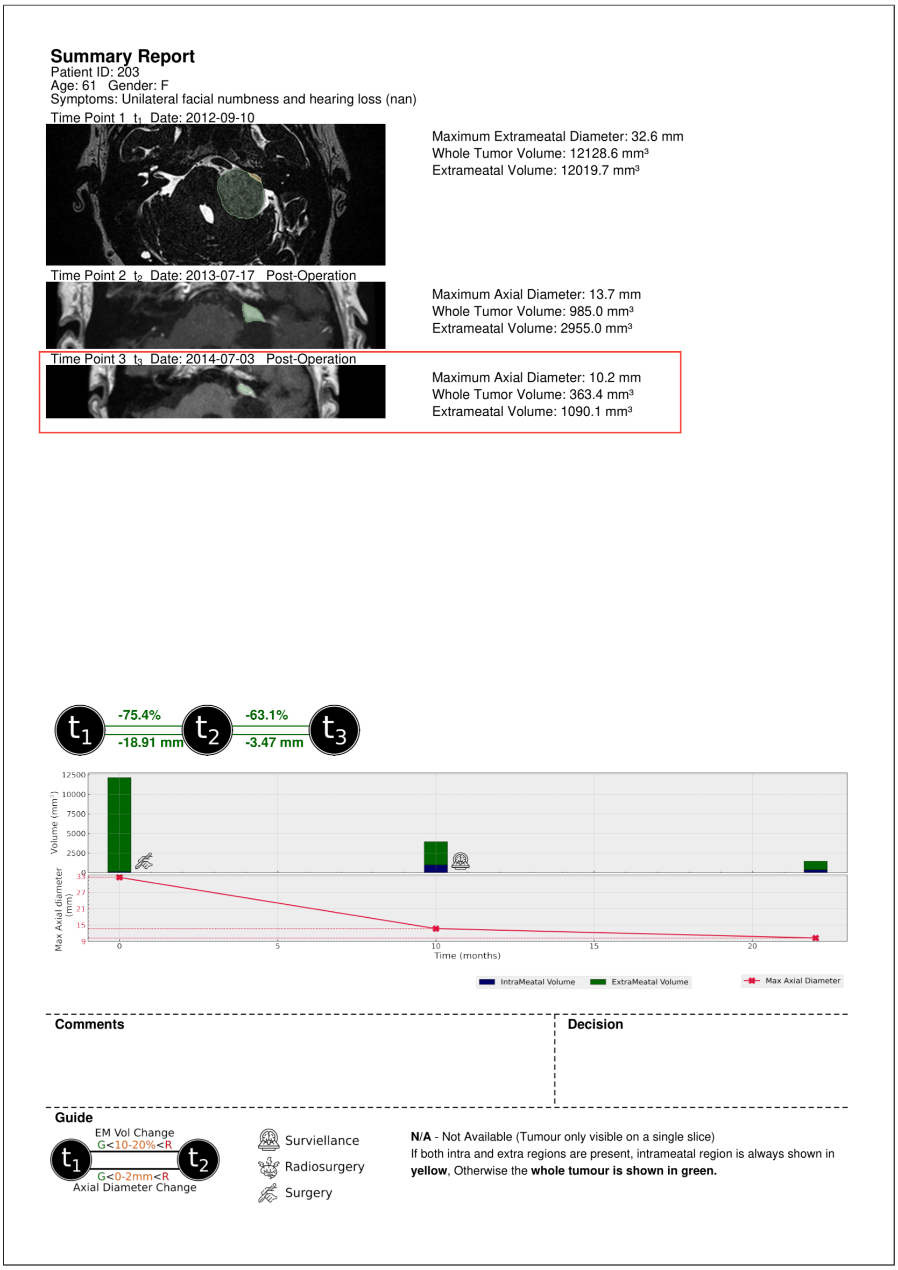

Fig. B3: The third time point segmentation was rejected (undersegmented) and therefore, the neuroradiologist manually extracted the linear measurement.

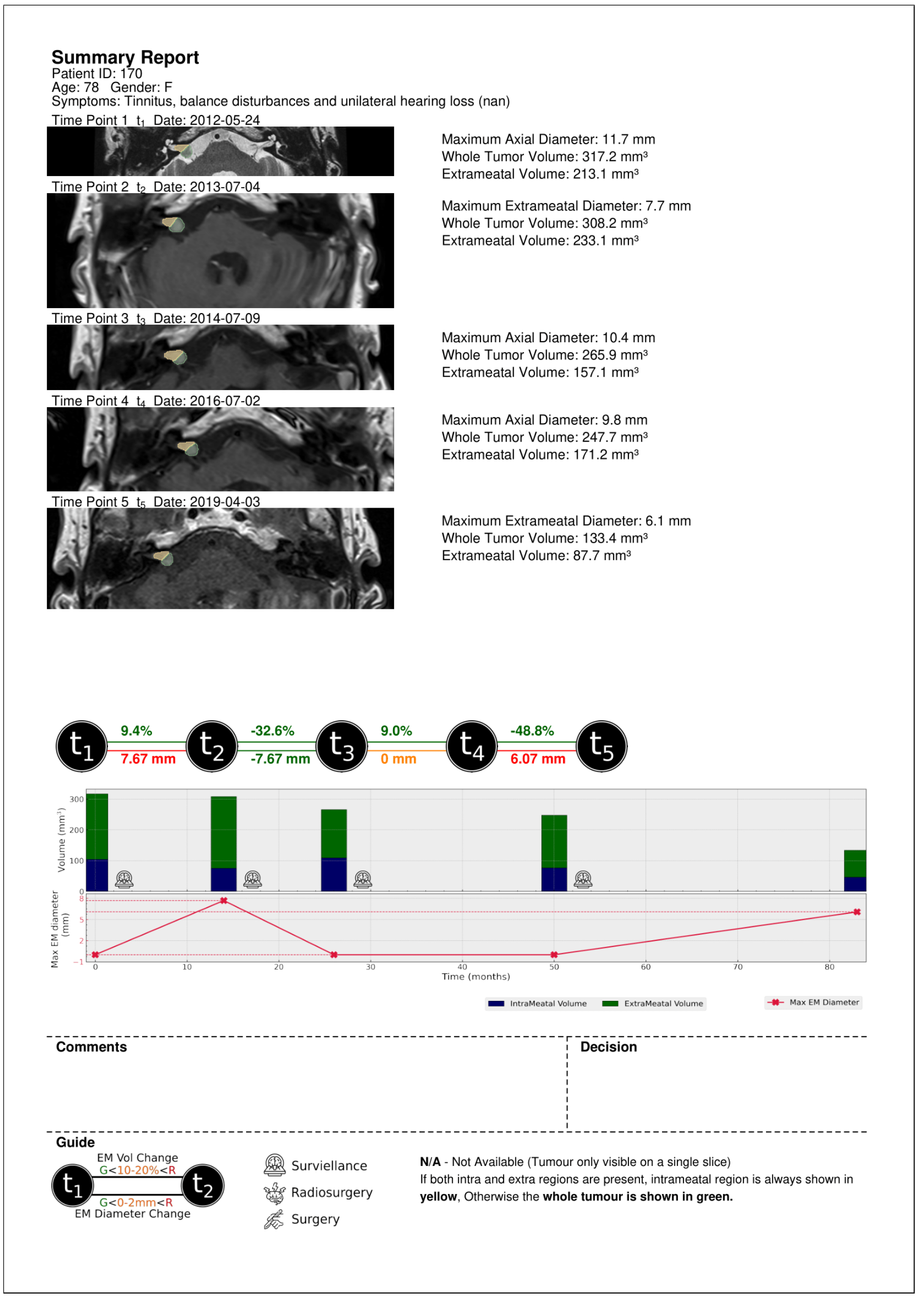

Fig. B4: This case was completely redone by the neuroradiologist. There is an inconsistency in the presented linear measurement and the intra-/extra-meatal boundaries were also rejected.

Workshop, MLCN 2022, Held in Conjunction with MICCAI 2022, Singapore, September 18, 2022, Proceedings, pp. 73–82 (2022). Springer

[5] Isensee, F., Jaeger, P.F., Kohl, S.A., Petersen, J., Maier-Hein, K.H.: nnu-net: a self-configuring method for deep learning-based biomedical image segmentation. Nature methods **18**(2), 203–211 (2021)

[6] Andrew, A.M.: Another efficient algorithm for convex hulls in two dimensions. Information Processing Letters **9**(5), 216–219 (1979)
