# Supplementary figures and images for: Artificial intelligence for personalized management of vestibular schwannoma: a multidisciplinary clinical implementation study
Source: JAMIA Open. 2026 Jan 6;9(1):ooaf163. doi: 10.1093/jamiaopen/ooaf163 (PMC12772638; doi:10.1093/jamiaopen/ooaf163)

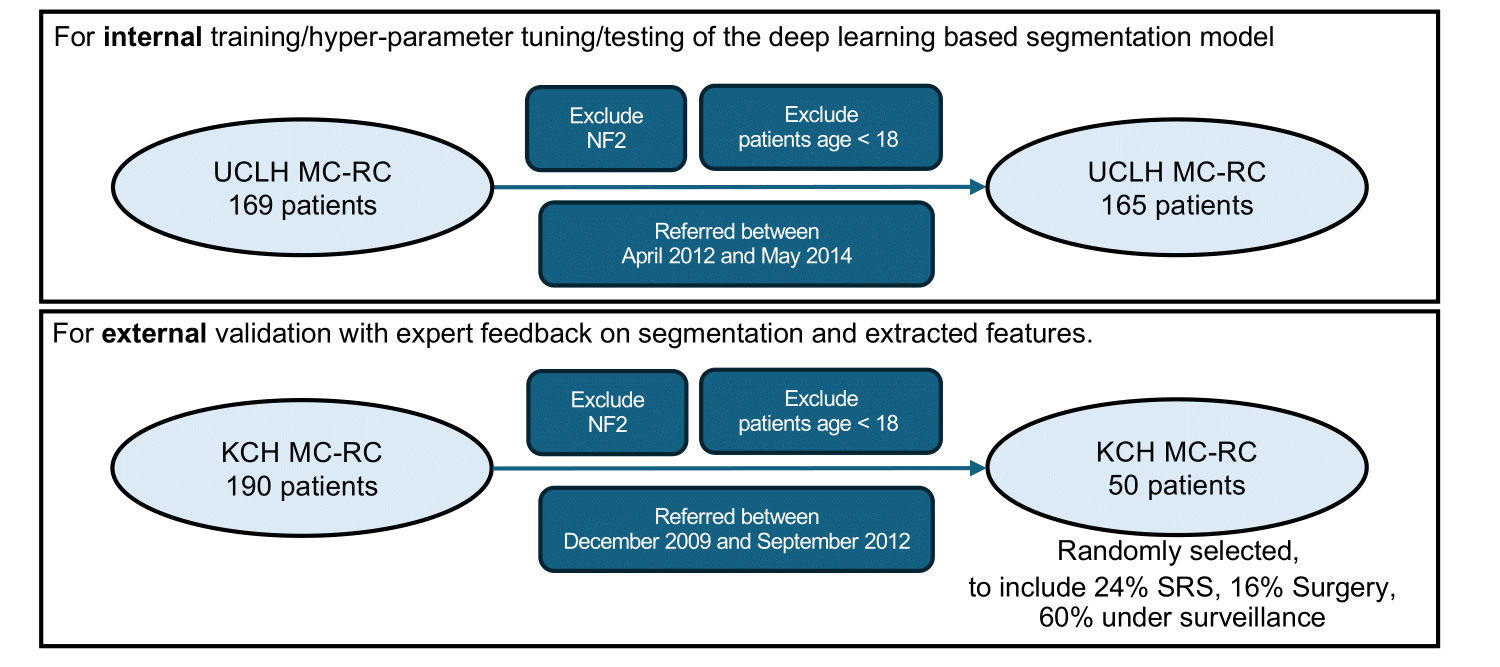

Supplement: ooaf163_Supplementary_Data [file ooaf163_supplementary_data.zip › MDM_Dataflow-1.png]

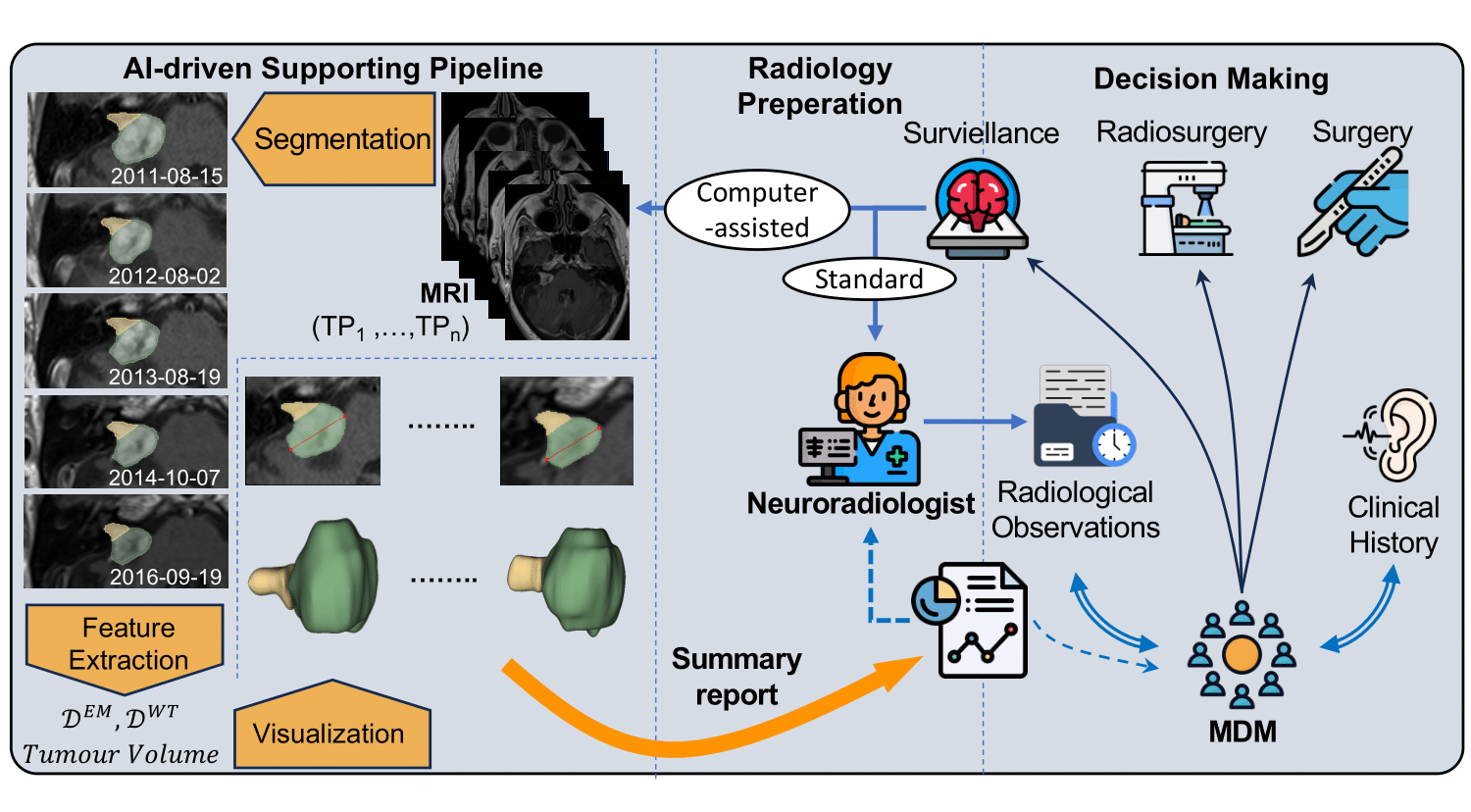

Supplement: ooaf163_Supplementary_Data [file ooaf163_supplementary_data.zip › outline-1.png]

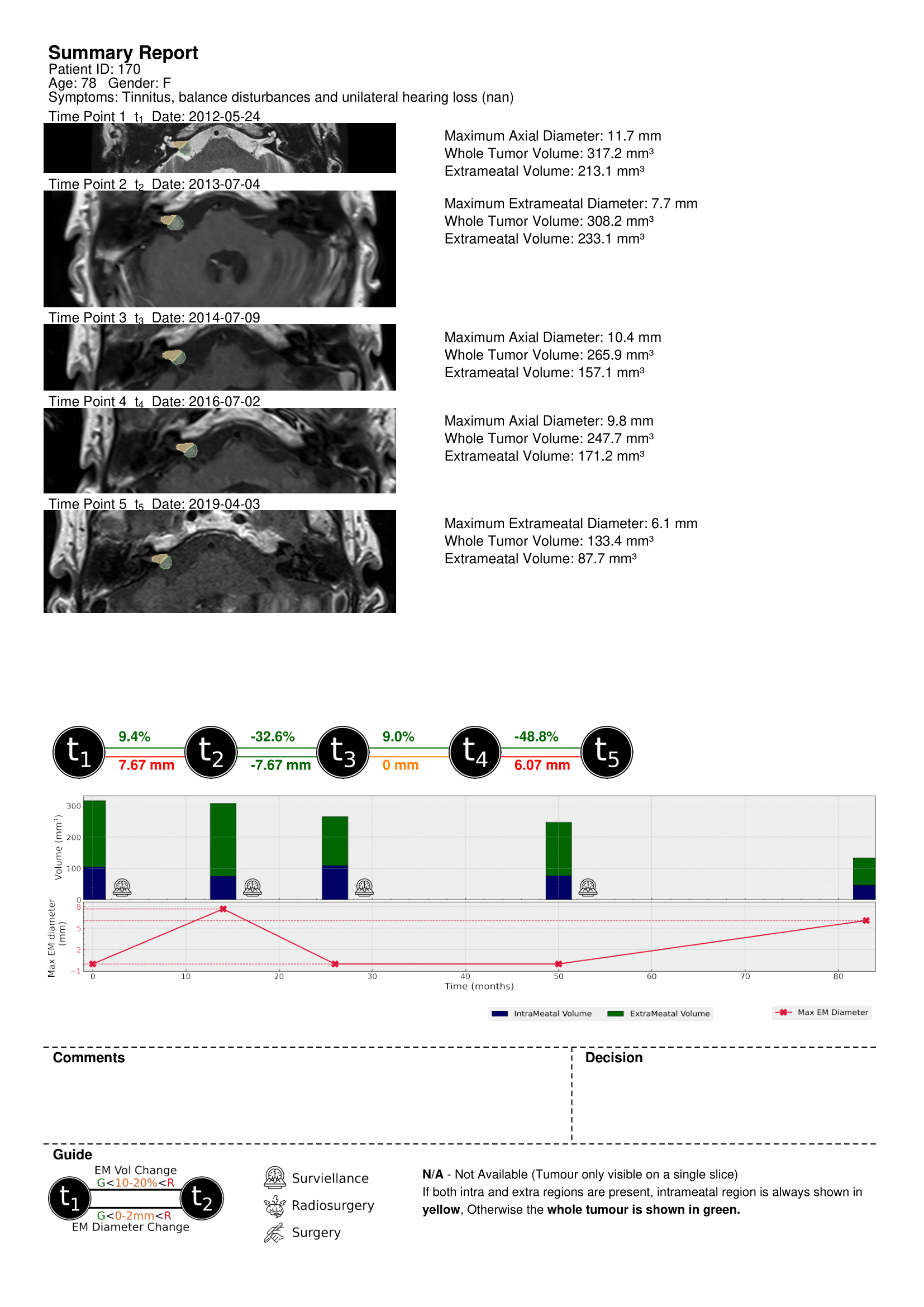

Supplement: ooaf163_Supplementary_Data [file ooaf163_supplementary_data.zip › summaryreport_170_rejected-1.png]

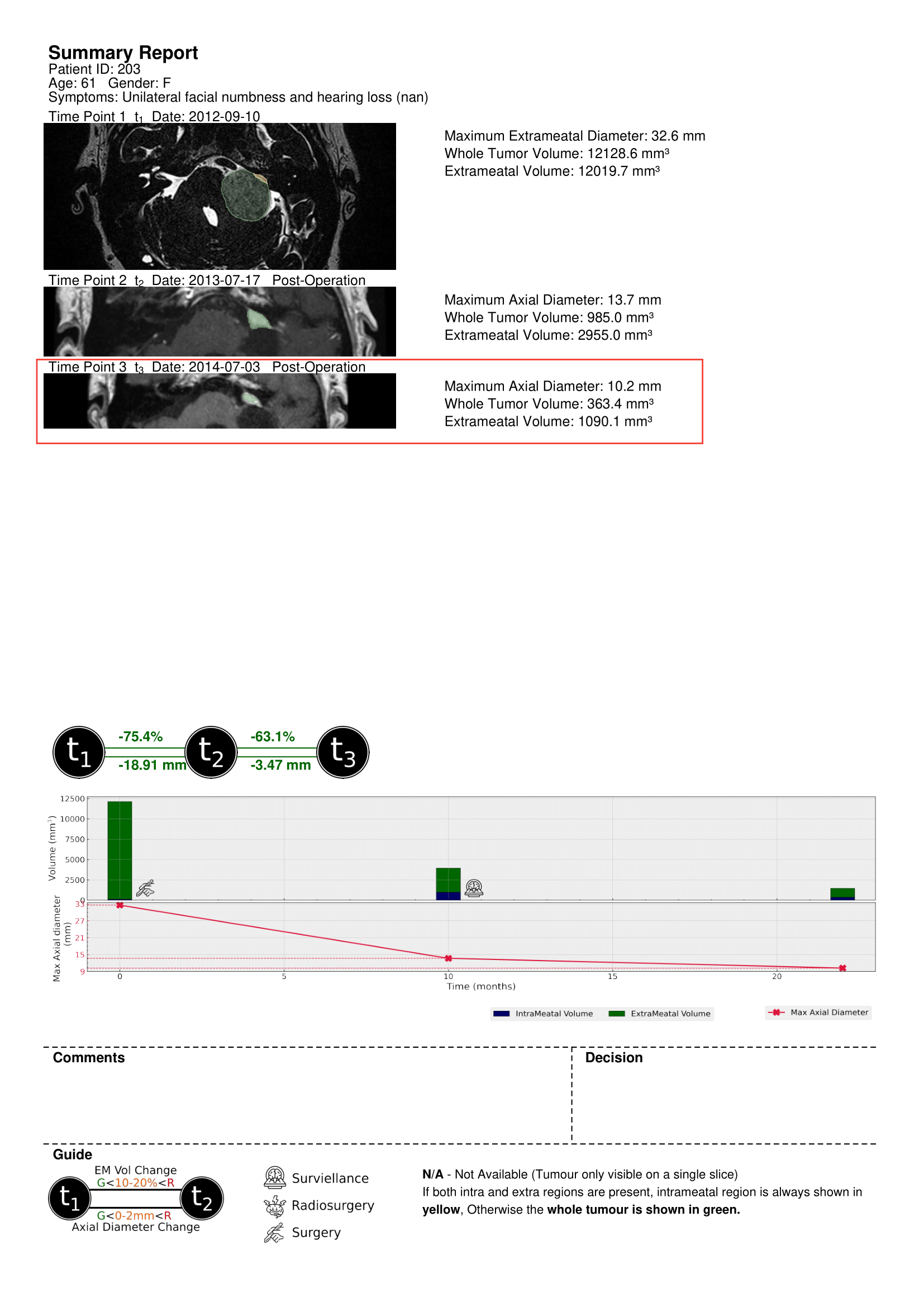

Supplement: ooaf163_Supplementary_Data [file ooaf163_supplementary_data.zip › summaryreport_203_undersegmented-1.png]

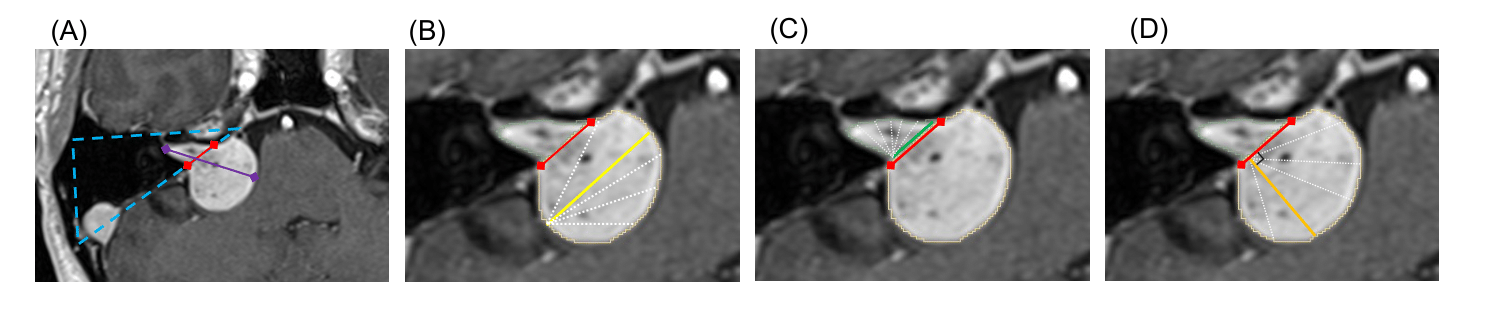

Supplement: ooaf163_Supplementary_Data [file ooaf163_supplementary_data.zip › additional_features_radiologyAI-1.png]
